# Supplementary material for: A Genetic Evaluation System for New Zealand White Rabbit Germplasm Resources Based on SSR Markers
Source: Animals (Basel). 2020 Jul 24;10(8):1258. doi: 10.3390/ani10081258 (PMC7460188; doi:10.3390/ani10081258)
Supplement: Supplementary file 1 [file animals-10-01258-s001.zip › animals-874418-supplementary/supplementary files/Table S1-2.docx]

Table S1. Combination of fluorescent SSR primers and reaction conditions

| Loci | primer sequence（5ʹ-3ʹ） | Lable | Position | *T_a_* (℃) |
| --- | --- | --- | --- | --- |
| SOL44 | GGCCCTAGTCTGACTCTGATTG | FAM | Chr14 | 58 |
|  | GGTGGGGCGGCGGGTCTGAAAC |  |  |  |
| 12L4A1 | GCTAATTACCCAAAGGAACATACA | HEX | Chr10 | 58 |
|  | CAGTGCAAATTTGGAAGGTCT |  |  |  |
| 6L1F10 | CAGAAGGCCATTTGTTTTG | TAMRA | Chr3 | 58 |
|  | GGTGATTCTTTCTTCTGCCTCTTA |  |  |  |
| 6L7C11 | ATGGCACGAATAATGTC | FAM | –– | 58 |
|  | TTTGGCAATACATGAGG |  |  |  |
| 6L3F8 | CTCCTGCCCTGTTCTAT | HEX | Chr4 | 55 |
|  | CAGGCTGGTCTTATTAC |  |  |  |
| 6L2H3 | CAAATGAAATGAGGAGGGAGATA | TAMRA | Chr3 | 58 |
|  | CATTGAGGACATTTGAGTAGTGAG |  |  |  |
| D3UTR2 | AGGAAGTGAGGGGAGGTGTT | FAM | Chr3 | 50 |
|  | ATAATGTGCTGCCAAAATAGAATA |  |  |  |
| 7L1B10 | TTGGCAGGAAGAAAAGGAAGATT | HEX | –– | 58 |
|  | TTTTTGTCATAAGCATTTGGGAAGTG |  |  |  |
| D6UTR4 | CAGAAGGGCATTTGTTTTG | TAMRA | Chr6 | 56 |
|  | GGTGATTCTTTCTTCTGCCTCTTA |  |  |  |
| SOL08 | GGATTGGGCCCTTTGCTCACACTTG | FAM | –– | 58 |
|  | ATCGCAGCCATATCTGAGAGAACTC |  |  |  |
| 12L1C2 | AGGGGCCTCCATCCTCTACA | HEX | –– | 56 |
|  | ATTATGTGTCAGGCAGGCTGTGTC |  |  |  |
| SAT12 | CTTGAGTTTAAATTCGGGC | TAMRA | Chr7 | 54 |
|  | GTTTGGATGCTATCTCAGTCC |  |  |  |
| 19L1C5 | AGTTGCTCCCACCCGATTTTA | FAM | –– | 56 |
|  | TGCTGTTGGGAGTAGATTGACC |  |  |  |
| SOL33 | GAAGGCTCTGAGATCTAGAT | HEX | Chr3 | 58 |
|  | GGGCCAATAGGTACTGATCCATGT |  |  |  |
| 12L5A6 | GGTGTGAACCACTAGATAGAA | TAMRA | –– | 58 |
|  | CAAAATTAGGTCCCTTGTAGT |  |  |  |
| SAT7 | GTAACCACCCCATGCACACT | FAM | Chr10 | 60 |
|  | GCACAATACCTGGGATGTAG |  |  |  |
| SOL03 | TACCGAGCACCAGATATTAGTTAC | HEX | –– | 54 |
|  | GTTACCTGTGTTTTGGAGTTCTTA |  |  |  |
| SOL30 | CCCGAGCCCCAGATATTGTTACCA | TAMRA | –– | 53 |
|  | TGCAGCTTCATAGTCTCAGGTC |  |  |  |
| 5LIE8 | CCAGCTGGTAATAGTAGAGA | FAM | –– | 50 |
|  | AAGGCATTTGTGGAGTGAA |  |  |  |
| 12LIE11 | AGTGGTAGCGCTTTGGTCTG | HEX | Chr12 | 54 |
|  | GCTCCTTGGGGCATTTG |  |  |  |
| SAT5 | GCTTCTGGCTTCAACCTGAC | TAMRA | Chr3 | 60 |
|  | CTTAGGGTGCAGAATTATAAGAG |  |  |  |
| L8B5 | TGTATGCCATGCTTTTAGTATT | FAM | –– | 60 |
|  | ATTTGCCCATTCTTTTGGTAT |  |  |  |

*T_a_* annealing temperature

Table S2. Combination of fluorescent SSR primers and reaction conditions

| Locus  Name | primer sequence（5’-3’） | Lable | Position | *T_a_* (℃) |
| --- | --- | --- | --- | --- |
| SAT8 | CAGACCCGGCAGTTGCAGAGGG | HEX | Chr17 | 53 |
|  | GAGAGAGGGATGGAGGTATG |  |  |  |
| D7UTR5 | ACACCTGGGGAATAAACAACAAG | TAMRA | Chr7 | 55 |
|  | GAGGGAGGCAGAGGGATAAGA |  |  |  |
| SAT3 | GGAGAGTGAATCAGTGGGTG | FAM | Chr3 | 56 |
|  | GAGGGAAAGAGAGAGACAGG |  |  |  |
| SAT4 | GGCCAGTGTCCTTACATTTGG | HEX | Chr15 | 53 |
|  | TGTTGCAGCGAATTGGGG |  |  |  |
| SOL62 | TGCCTTTAGGATTGGTCTATCTCTG | TAMRA | –– | 61 |
|  | GCGGGAGAGGGGGAGAGGGGGAGAG |  |  |  |
| SAT2 | GCTCTCCTTTGGCATACTCC | FAM | Chr6 | 54 |
|  | GCTTTGGATAGGCCCAGATC |  |  |  |
| INRACCD  DV0003 | GATCAGCGAGCGCCTCTC | HEX | Chr16 | 58 |
|  | TCCATCTGAATGAGGCACAA |  |  |  |
| INRACCD  DV0007 | CTGCTAGCTCTGGGTGGAAG | TAMRA | Chr5 | 60 |
|  | TGTGTGACCTTGTGGCCTTA |  |  |  |
| INRACCD  DV0010 | GAACCAGCAAATGGAAACTCA | FAM | Chr9 | 53 |
|  | CCAAGGTCTGAAAGTCATTGC |  |  |  |
| INRACCD  DV0087 | GATCTGGGACTCCAGAGTGTG | HEX | Chr8 | 62 |
|  | GAACACCGGTCTGGATGG |  |  |  |
| INRACCD  DV0108 | GTTCACTTTTGCTTGCCAGTT | TAMRA | Chr11 | 56 |
|  | TCTGCAGGCATCCACTAACTT |  |  |  |
| INRACCD  DV0152 | CCAGGCTCTTGCCTCTTATCT | FAM | Chr17 | 62 |
|  | ACTCTCCTCTCGCCTCTCACT |  |  |  |
| INRACCD  DV0185 | GTTCACTCAAAGTAACTGACAAGA | HEX | Chr16 | 53 |
|  | TCCATTCAGCAACCAATGAA |  |  |  |
| INRACCD  DV0190 | GCCAATACCAAAAACAGCAAC | TAMRA | Chr18 | 56 |
|  | TGGGAGAGCTAAGAAAAGTCG |  |  |  |
| INRACCD  DV0192 | TGCAATAGGTGGAGGCTTAGA | FAM | Chr2 | 58 |
|  | TCCACAGAGGAGATATAGTGGTCTT |  |  |  |
| INRACCD  DV0309 | CAGAGAGAAGGAGAGGCAGAA | TAMRA | Chr19 | 61 |
|  | GCCGTTGCAGCCAGTTAC |  |  |  |
| INRACCD  DV0313 | TGTGATACCCCGGAGTGTTT | FAM | Chr14 | 61 |
|  | TCGCTTGACTTCTTGGGTCT |  |  |  |
| INRACCD  DV0314 | CCTGTAGTAATACCGCCCACA | HEX | Chr4 | 62 |
|  | CGGGAGACAGGTATGGAGTT |  |  |  |
| INRACCD  DV0346 | ATGGAGGGTTTCTCCCCAAT | FAM | Chr13 | 61 |
|  | CAGCCAAATAGCCACAGTCA |  |  |  |
| INRACCD  DV0160 | CCAGAACTTTCCAGACAGCAT | HEX | Chr9 | 56 |
|  | CCTAAAACGCTGTCATCCTGA |  |  |  |
| INRACCD  DV0157 | AACACTTGCCCCTCTTTTCAT | TAMRA | Chr8 | 56 |
|  | CAGGTTGTGGGAGTTCTTGTC |  |  |  |
