# Supplementary material for: A Genetic Evaluation System for New Zealand White Rabbit Germplasm Resources Based on SSR Markers
Source: Animals (Basel). 2020 Jul 24;10(8):1258. doi: 10.3390/ani10081258 (PMC7460188; doi:10.3390/ani10081258)
Supplement: Supplementary file 1 [file animals-10-01258-s001.zip › animals-874418-supplementary/supplementary files/Table S3-5.docx]

Table S3 A list of selected loci.

| Number | Loci | Number | Loci | Number | Loci |
| --- | --- | --- | --- | --- | --- |
| 1 | L8B5 | 16 | INRACCDDV0152 | 31 | D6UTR4 |
| 2 | SAT8 | 17 | INRACCDDV0309 | 32 | SOL08 |
| 3 | D7UTR5 | 18 | INRACCDDV0313 | 33 | 12L1C2 |
| 4 | SAT3 | 19 | INRACCDDV0314 | 34 | SAT12 |
| 5 | SAT4 | 20 | INRACCDDV0346 | 35 | 19L1C5 |
| 6 | SOL62 | 21 | INRACCDDV0160 | 36 | SOL33 |
| 7 | SAT2 | 22 | INRACCDDV0157 | 37 | 12L5A6 |
| 8 | INRACCDDV0003 | 23 | SOL44 | 38 | SAT7 |
| 9 | INRACCDDV0007 | 24 | 12L4A1 | 39 | SOL03 |
| 10 | INRACCDDV0010 | 25 | 6L1F10 | 40 | SOL30 |
| 11 | INRACCDDV0087 | 26 | SAT13 | 41 | 5LIE8 |
| 12 | INRACCDDV0018 | 27 | 6L3F8 | 42 | 12LIE11 |
| 13 | INRACCDDV0192 | 28 | 6L2H3 | 43 | SAT5 |
| 14 | INRACCDDV0185 | 29 | D3UTR2 |  |  |
| 15 | INRACCDDV0190 | 30 | 7L1B10 |  |  |

Table S4 No. 1 to 15 SSR marker combination information.

| C  1 | C  2 | C  3 | C  4 | C  5 | C  6 | C  7 | C  8 | C  9 | C  10 | C  11 | C  12 | C  13 | C  14 | C  15 |
| --- | --- | --- | --- | --- | --- | --- | --- | --- | --- | --- | --- | --- | --- | --- |
| 3 | 1 | 2 | 2 | 1 | 2 | 2 | 6 | 2 | 5 | 8 | 1 | 2 | 3 | 1 |
| 5 | 3 | 5 | 4 | 2 | 4 | 4 | 7 | 5 | 9 | 9 | 5 | 3 | 4 | 3 |
| 6 | 6 | 6 | 5 | 5 | 5 | 8 | 11 | 7 | 10 | 10 | 7 | 4 | 5 | 6 |
| 7 | 7 | 7 | 7 | 8 | 6 | 15 | 12 | 9 | 12 | 11 | 9 | 5 | 10 | 8 |
| 11 | 9 | 9 | 10 | 9 | 9 | 16 | 13 | 10 | 13 | 17 | 10 | 6 | 13 | 9 |
| 12 | 11 | 10 | 13 | 11 | 14 | 17 | 14 | 11 | 19 | 18 | 14 | 8 | 15 | 10 |
| 13 | 13 | 11 | 14 | 12 | 15 | 18 | 15 | 12 | 20 | 20 | 15 | 9 | 17 | 12 |
| 14 | 14 | 13 | 16 | 15 | 16 | 20 | 17 | 15 | 23 | 22 | 18 | 10 | 18 | 14 |
| 15 | 15 | 17 | 17 | 16 | 20 | 21 | 18 | 17 | 24 | 23 | 19 | 14 | 19 | 15 |
| 16 | 20 | 18 | 18 | 17 | 21 | 22 | 19 | 18 | 26 | 25 | 20 | 15 | 20 | 17 |
| 17 | 21 | 19 | 23 | 18 | 22 | 23 | 22 | 20 | 28 | 26 | 22 | 16 | 21 | 18 |
| 22 | 22 | 21 | 25 | 19 | 23 | 24 | 25 | 21 | 29 | 28 | 25 | 20 | 23 | 21 |
| 24 | 23 | 22 | 26 | 21 | 24 | 25 | 30 | 23 | 30 | 29 | 26 | 21 | 26 | 23 |
| 26 | 24 | 24 | 27 | 22 | 25 | 26 | 31 | 24 | 31 | 32 | 29 | 23 | 29 | 24 |
| 27 | 25 | 25 | 29 | 23 | 26 | 27 | 32 | 25 | 33 | 33 | 31 | 26 | 31 | 26 |
| 29 | 27 | 27 | 30 | 24 | 29 | 28 | 34 | 28 | 35 | 34 | 33 | 27 | 32 | 30 |
| 32 | 29 | 31 | 31 | 26 | 31 | 29 | 35 | 30 | 37 | 36 | 34 | 28 | 34 | 31 |
| 34 | 30 | 32 | 32 | 28 | 33 | 31 | 36 | 34 | 38 | 37 | 35 | 31 | 36 | 34 |
| 37 | 35 | 34 | 36 | 33 | 34 | 34 | 38 | 35 | 39 | 39 | 36 | 33 | 38 | 35 |
| 39 | 36 | 35 | 37 | 36 | 37 | 36 | 39 | 36 | 40 | 40 | 37 | 35 | 39 | 38 |
| 41 | 39 | 38 | 38 | 40 | 38 | 38 | 40 | 38 | 41 | 41 | 38 | 37 | 40 | 39 |
| 42 | 42 | 40 | 40 | 43 | 40 | 39 | 41 | 42 | 42 | 43 | 42 | 39 | 42 | 42 |

Table S5 No. 16 to 30 SSR marker combination information.

| C  16 | C  17 | C  18 | C  19 | C  20 | C  21 | C  22 | C  23 | C  24 | C  25 | C  26 | C  27 | C  28 | C  29 | C  30 |
| --- | --- | --- | --- | --- | --- | --- | --- | --- | --- | --- | --- | --- | --- | --- |
| 1 | 10 | 3 | 1 | 1 | 1 | 2 | 3 | 2 | 1 | 2 | 2 | 3 | 1 | 2 |
| 5 | 11 | 5 | 3 | 2 | 2 | 3 | 4 | 6 | 2 | 4 | 3 | 4 | 10 | 4 |
| 8 | 12 | 6 | 5 | 5 | 4 | 4 | 6 | 7 | 5 | 5 | 4 | 6 | 11 | 5 |
| 9 | 13 | 7 | 6 | 6 | 5 | 6 | 10 | 12 | 6 | 6 | 7 | 7 | 14 | 6 |
| 10 | 14 | 8 | 7 | 7 | 6 | 8 | 11 | 13 | 7 | 9 | 8 | 10 | 19 | 8 |
| 11 | 15 | 10 | 11 | 10 | 7 | 11 | 14 | 14 | 8 | 11 | 11 | 11 | 20 | 10 |
| 12 | 22 | 11 | 13 | 15 | 9 | 13 | 16 | 15 | 10 | 13 | 12 | 12 | 21 | 12 |
| 16 | 23 | 12 | 15 | 17 | 10 | 14 | 20 | 16 | 11 | 14 | 13 | 13 | 22 | 16 |
| 17 | 24 | 13 | 17 | 18 | 12 | 15 | 22 | 17 | 13 | 15 | 15 | 16 | 23 | 18 |
| 18 | 25 | 14 | 21 | 19 | 14 | 17 | 25 | 20 | 14 | 16 | 18 | 17 | 26 | 19 |
| 20 | 27 | 15 | 22 | 23 | 16 | 19 | 26 | 27 | 17 | 19 | 19 | 18 | 27 | 20 |
| 24 | 28 | 20 | 23 | 24 | 18 | 21 | 27 | 29 | 19 | 20 | 20 | 19 | 28 | 22 |
| 25 | 29 | 24 | 24 | 26 | 19 | 22 | 28 | 30 | 20 | 21 | 24 | 23 | 31 | 23 |
| 27 | 30 | 26 | 26 | 27 | 21 | 23 | 29 | 31 | 21 | 22 | 25 | 24 | 32 | 28 |
| 28 | 31 | 27 | 28 | 28 | 25 | 26 | 31 | 33 | 22 | 23 | 26 | 26 | 33 | 31 |
| 29 | 33 | 29 | 29 | 33 | 26 | 27 | 33 | 34 | 26 | 26 | 29 | 27 | 34 | 33 |
| 35 | 34 | 32 | 30 | 34 | 30 | 29 | 35 | 36 | 28 | 27 | 30 | 28 | 35 | 36 |
| 36 | 35 | 34 | 33 | 35 | 31 | 31 | 37 | 37 | 32 | 31 | 38 | 31 | 36 | 37 |
| 38 | 36 | 36 | 34 | 37 | 34 | 32 | 38 | 38 | 37 | 34 | 39 | 33 | 38 | 38 |
| 41 | 39 | 37 | 37 | 39 | 35 | 35 | 39 | 39 | 41 | 39 | 40 | 36 | 39 | 39 |
| 42 | 40 | 38 | 41 | 40 | 36 | 36 | 40 | 42 | 42 | 40 | 41 | 41 | 40 | 40 |
| 43 | 42 | 41 | 43 | 41 | 37 | 43 | 41 | 43 | 43 | 41 | 42 | 42 | 43 | 41 |

Table S3. No. 16 to 30 marker loci combination information table

| C  16 | C  17 | C  18 | C  19 | C  20 | C  21 | C  22 | C  23 | C  24 | C  25 | C  26 | C  27 | C  28 | C  29 | C  30 |
| --- | --- | --- | --- | --- | --- | --- | --- | --- | --- | --- | --- | --- | --- | --- |
| 1 | 10 | 3 | 1 | 1 | 1 | 2 | 3 | 2 | 1 | 2 | 2 | 3 | 1 | 2 |
| 5 | 11 | 5 | 3 | 2 | 2 | 3 | 4 | 6 | 2 | 4 | 3 | 4 | 10 | 4 |
| 8 | 12 | 6 | 5 | 5 | 4 | 4 | 6 | 7 | 5 | 5 | 4 | 6 | 11 | 5 |
| 9 | 13 | 7 | 6 | 6 | 5 | 6 | 10 | 12 | 6 | 6 | 5 | 7 | 14 | 6 |
| 10 | 14 | 8 | 7 | 7 | 6 | 8 | 11 | 13 | 7 | 9 | 8 | 10 | 20 | 8 |
| 11 | 15 | 10 | 9 | 10 | 7 | 11 | 14 | 14 | 8 | 11 | 11 | 11 | 21 | 10 |
| 12 | 17 | 11 | 13 | 13 | 9 | 13 | 16 | 15 | 10 | 13 | 12 | 12 | 22 | 12 |
| 16 | 21 | 12 | 15 | 17 | 10 | 14 | 17 | 16 | 11 | 14 | 13 | 13 | 23 | 16 |
| 17 | 24 | 13 | 18 | 18 | 12 | 15 | 22 | 17 | 13 | 15 | 15 | 16 | 24 | 17 |
| 18 | 25 | 14 | 19 | 19 | 14 | 17 | 24 | 18 | 14 | 16 | 19 | 18 | 25 | 20 |
| 19 | 26 | 15 | 23 | 20 | 16 | 18 | 27 | 21 | 18 | 20 | 20 | 19 | 28 | 21 |
| 22 | 27 | 17 | 24 | 25 | 19 | 21 | 28 | 22 | 20 | 21 | 21 | 20 | 29 | 22 |
| 26 | 29 | 22 | 25 | 26 | 20 | 23 | 29 | 29 | 21 | 22 | 22 | 21 | 30 | 24 |
| 27 | 30 | 26 | 26 | 28 | 21 | 24 | 30 | 31 | 22 | 23 | 26 | 25 | 33 | 25 |
| 29 | 31 | 28 | 28 | 29 | 23 | 25 | 31 | 32 | 23 | 24 | 27 | 26 | 34 | 30 |
| 30 | 32 | 29 | 30 | 30 | 27 | 28 | 33 | 33 | 24 | 25 | 28 | 28 | 35 | 33 |
| 31 | 33 | 31 | 31 | 35 | 28 | 29 | 35 | 36 | 28 | 28 | 31 | 29 | 36 | 35 |
| 37 | 35 | 34 | 32 | 36 | 29 | 31 | 37 | 38 | 30 | 29 | 32 | 30 | 37 | 38 |
| 38 | 36 | 36 | 35 | 37 | 32 | 33 | 39 | 39 | 34 | 33 | 40 | 33 | 38 | 39 |
| 40 | 37 | 37 | 36 | 39 | 36 | 34 | 40 | 40 | 39 | 36 | 41 | 35 | 40 | 40 |
| 43 | 38 | 38 | 39 | 41 | 37 | 37 | 41 | 41 | 43 | 41 | 42 | 38 | 41 | 41 |
| 44 | 41 | 39 | 43 | 42 | 38 | 38 | 42 | 44 | 44 | 42 | 43 | 43 | 42 | 42 |
| 45 | 42 | 40 | 45 | 44 | 39 | 45 | 43 | 45 | 45 | 43 | 44 | 44 | 45 | 43 |
