# Supplementary figures and images for: A Genetic Evaluation System for New Zealand White Rabbit Germplasm Resources Based on SSR Markers
Source: Animals (Basel). 2020 Jul 24;10(8):1258. doi: 10.3390/ani10081258 (PMC7460188; doi:10.3390/ani10081258)

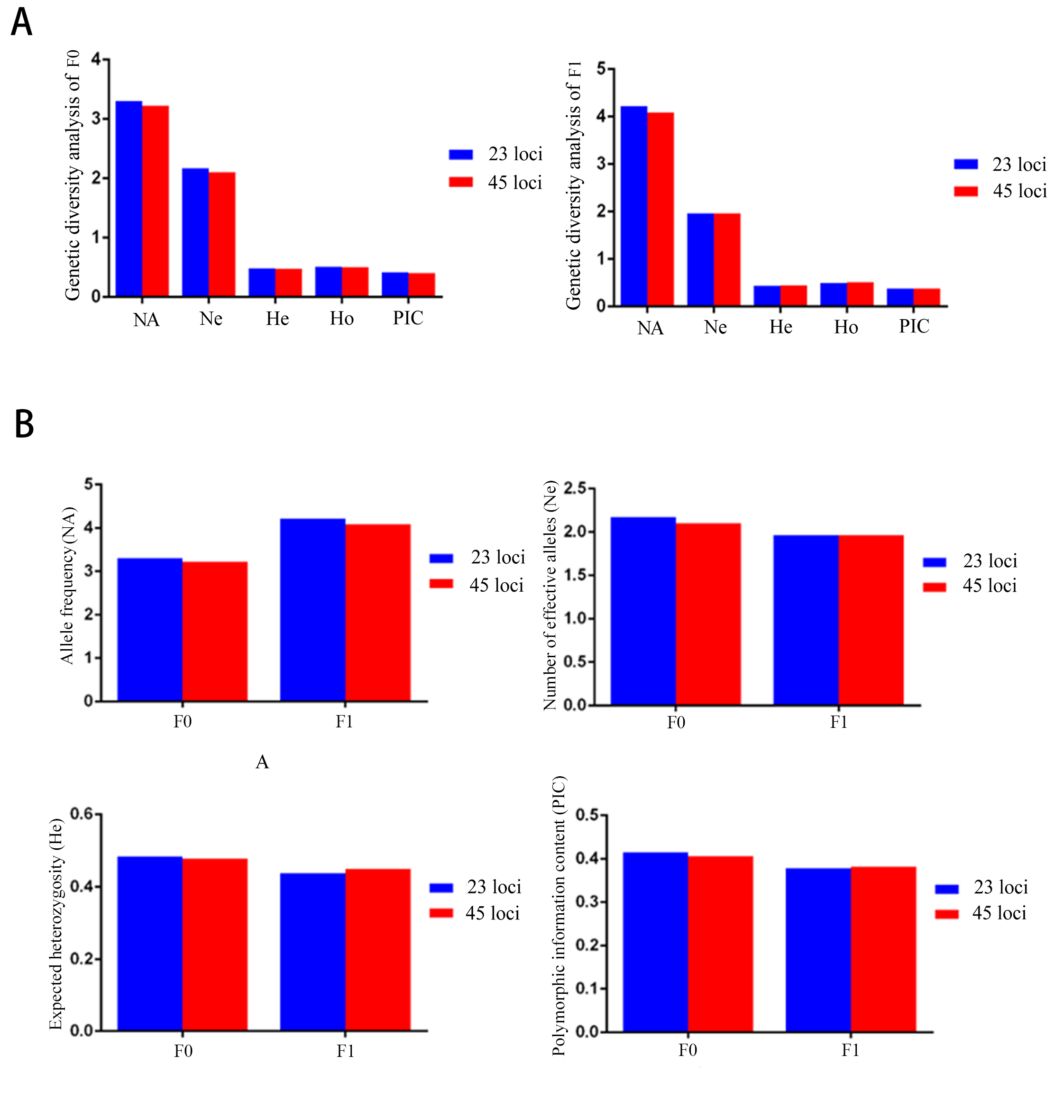

Supplement: Supplementary file 1 [file animals-10-01258-s001.zip › animals-874418-supplementary/supplementary files/Fig.S3.tif]
